# Supplementary material for: Turning off the tap: Common domestic water conservation actions insufficient to alleviate drought in the United States of America
Source: PLoS One. 2020 Mar 4;15(3):e0229798. doi: 10.1371/journal.pone.0229798 (PMC7055883; doi:10.1371/journal.pone.0229798)
Supplement: S1 Table — (PDF) [file pone.0229798.s001.pdf]

Supplementary Information for:

Turning off the Tap: Common domestic water conservation actions insufficient to alleviate drought in the United States of America

Diana M Ruiz
Heather Tallis
Bernie R Tershy
Donald A Croll

Email: dmadriga@ucsc.edu

This PDF file includes:
Supplementary text
SI References

SI1. Commonly Recommended Water Conservation Actions in the U.S. and Associated Estimated Household Savings in 2010

Action	Average Water Savings (G^{-1} $household^{-1}$ month)	Estimated Savings Comments	Reference
Repairs and Replacements			
Repairing leaky faucet	8.67	estimate is per household with 3 faucets dripping at 1 drip per minute, assumes all households have 3 leaky faucets	Howard Perlman, 2014, The USGS Water Sciecne School, Drip Calculator
Repairing household leaks	833.33	estimate is average household leaks	WaterSense, U.S. Environmental Protection Agency, Office of Wastwater Management 4204M. 2014. WaterSense. Fix A Leak Week.
Install WaterSense showerheads	191.67	estimate is average household savings, assumes all households install WaterSense labled showerheads: OVERESTIMATE (households that already have WaterSense)	WaterSense, U.S. Environmental Protection Agency, Office of Wastwater Management 4204M. 2014. WaterSense. Fix A Leak Week.
Install WaterSense bathroom faucet & aerators	41.67	estimate is average household savings, assumes all households install WaterSense labled faucet & aerators: OVERESTIMATE (households that already have WaterSense)	WaterSense, U.S. Environmental Protection Agency, Office of Wastwater Management 4204M. 2014. WaterSense. WaterSense Labeled Bathroom Faucet Fact Sheet
Install WaterSense labeled low flow toilets	457.01	estimate is average household savings if all inefficient toilets were replaced by WaterSense labeled toilets	WaterSense, U.S. Environmental Protection Agency, Office of Wastwater Management 4204M. 2014. WaterSense.WaterSense Labeled Toilets
Install ENERGYSTAR dishwasher	83.43	Unknown how many households require upgrade	Alliance for Water Efficiency. 2011. Home Water Works, Dish Washer
Install ENERGYSTAR clothes washer	113.97	Estimate is savings for replacing all existing	Clothes washer, Products, ENERGY STAR, U.S.

		inefficient clothes washers per household	Environmental Protection Agency 2014
Install WaterSense irrigation controller	85.69	estimate is savings of replacing current irrigation systems	U.S. Environmental Protection Agency. 2013. WaterSense. Fact Sheet Irrigation Controllers508. EPA-832-F-11-001
Behaviors			
Close faucet and use a bowl of water when hand washing or rinsing dishes	12.11	Compared to running faucet	South Staffs Water. 2010. Water use in your home. 1938_3/2010
Close faucet when brushing teeth	619.20	estimate includes total population including children under 1 year old	U.S. Environmental Protection Agency. 2012. Green Homes, bathroom
Close faucet and fill sink with a few inches of water when shaving	88.00	Estimate is savings per household by men over 18, assumes all men over 18 shave daily OVERESTIMATE (not all men over 18 shave daily)	South Carolina Department of Nautal Resources.2012. ACR Basin National Estuarine Research Reserve. How Many Gallons Can You Save?
Only full loads in dishwasher	105.31	estimate is savings per household with a dishwasher, based on combined average savings of both dish and clothes washer corrected with proportion of water use (dish to clothes) includes only households with a dishwasher	South Staffs Water. 2010. Water Usage
Only full loads in dishwasher, washing machine	349.37	estimate is savings per household with a clothes washer, based on combined average savings of both dish and clothes washer corrected with proportion of water use (clothes to dish) includes only households with a clothes washer	South Staffs Water. 2010. Water Usage
Take short (5 minute) showers instead of long showers (8 minutes)	774.00	Assumes everyone intially took a long shower each day and that no one ever takes	South Staffs Water. 2010. Water Usage

		a bath UNDERESTIMATE (many already take short showers and savings relative to baths are significantly larger)	
**Total Gallons Saved Per Household Per Month**	3763.42		
