# Supplementary material for: Turning off the tap: Common domestic water conservation actions insufficient to alleviate drought in the United States of America
Source: PLoS One. 2020 Mar 4;15(3):e0229798. doi: 10.1371/journal.pone.0229798 (PMC7055883; doi:10.1371/journal.pone.0229798)
Supplement: Attachment — Submitted filename: Response to Reviewers .docx [file pone.0229798.s003.docx]

**Response to Reviewers**

Turning off the tap: Common domestic water conservation actions insufficient to alleviate drought in the United States of America

PONE-D-19-28748

Reviewer comment:

*“****Please add some your Options for the Future: Balancing Water Demand and Water Resources at domestic level***

*Please estimated water savings for a house with low pressure in county then compare to a house with high pressure,”*

**Response:**

**We are unclear as to the reviewer’s use of the term “pressure” here, and so respond on the basis of two possible interpretations. The reviewer may be using pressure to refer to water pressure entering the household through pipes from the municipality or well. In this case, it would be expected that a household with high water pressure would use more water than a low water pressure household, and so water saving devices may have larger benefit when installed in high water pressure households. To analyze this variation, we would need to know average water pressure in each county, and make an assumption about how many houses already have pressure regulating devices installed. Water pressure can vary dramatically even within the service area of one municipal water provider (based on distance from source, elevation, pipe diameter, etc). We were unable to identify an available data source that would allow us to robustly estimate impact of water pressure on adoption of other water saving devices. We have added text to the methods section clarifying this limitation, lines 152-155.**

**A second interpretation of “pressure” in the reviewer’s comment could relate to household water demand, wherein high pressure would represent households with high water demand. Our methods relied on total domestic demand reported at the county level. We are not aware of data avialable at the individual household level and so are unable to make household level demand comparisons.**

Reviewer comment:

**“your data will be more reliable for future estimation if you** please add some data regarding important voluntary domestic water conservation measures include the following:

- Limiting toilet flushing.
- Adopting water-saving plumbing fixtures, such as toilets and shower heads.
- Adopting water-efficient appliances (notably washing machines).
- Limiting outdoor uses of water, as by watering lawns and gardens during the evening and early morning, and washing cars on lawns without using a hose.”

**Response:**

**Our chosen water saving interventions include all elements suggested here, except limiting toilet flushing. Our original manuscript includes water saving plumbing fixtures as specified for toilets (in Table 1, row 4 “Install WaterSense labeled low flow toilets”), showerheads (Table 1, row 5 “Install WaterSense showerheads”) and washing machines (Table 1, row 6 “Install ENERGYSTAR clothes washer”). We also include the commonly recommended action to limit outdoor water use as the installation of a WaterSense irrigation controller (Table 1, row 9). WaterSense irrigation controllers earn the WaterSense label by meeting EPA standards for avoiding overwatering. These irrigation controls assess actual local weather conditions to determine watering needs rather than preset timers. We feel this adequately addresses the action of efficient outdoor watering in the most reliably measured manner.** **All of the mentioned actions and the derivation of their estimated savings are also found in Supplemental Table 1.**

**As washing cars on lawns and limiting toilet flushing were not found to be a commonly recommended action and difficult to reliably estimate potential savings, we have not included it in our analysis. However, we have added text to note such exclusions in our methods, lines 133-140.**
