# Supplementary material for: Turning off the tap: Common domestic water conservation actions insufficient to alleviate drought in the United States of America
Source: PLoS One. 2020 Mar 4;15(3):e0229798. doi: 10.1371/journal.pone.0229798 (PMC7055883; doi:10.1371/journal.pone.0229798)
Supplement: Attachment — Submitted filename: Reviewer comments.docx [file pone.0229798.s002.docx]

**Reviewer comments**

Turning off the tap: Common domestic water conservation actions insufficient to alleviate drought in the United States of America

PONE-D-19-28748

**Please add some your Options for the Future: Balancing Water Demand and Water Resources at domestic level**

Please estimated water savings for a house with low pressure in county then compare to a house with high pressure, **your data will be more reliable for future estimation if you** please add some data regarding important voluntary domestic water conservation measures include the following:

- Limiting toilet flushing.
- Adopting water-saving plumbing fixtures, such as toilets and shower heads.Bottom of Form
- Adopting water-efficient appliances (notably washing machines).
- Limiting outdoor uses of water, as by watering lawns and gardens during the evening and early morning, and washing cars on lawns and without using a hose.
